# Medical Cannabis for Chronic Noncancer Pain: A Systematic Review of Health Care Recommendations

**DOI:** 10.1155/2021/8857948

**Published:** 2021-02-04

**Authors:** Yaping Chang, Meng Zhu, Christopher Vannabouathong, Raman Mundi, Roland S. Chou, Mohit Bhandari

**Affiliations:** ^1^OrthoEvidence Inc., Burlington, ON, Canada; ^2^Department of Health Research Methods, Evidence & Impact, McMaster University, Hamilton, ON, Canada; ^3^Department of Surgery, University of Toronto, Toronto, ON, Canada; ^4^Sunnybrook Holland Orthopaedic and Arthritic Centre, Toronto, ON, Canada; ^5^Faculty of Health Sciences, McMaster University, Hamilton, ON, Canada; ^6^Department of Surgery, McMaster University, Hamilton, ON, Canada

## Abstract

**Purpose:**

Medical cannabis for patients with chronic noncancer pain (CNCP) has been the focus of numerous health care recommendations. We conducted a systematic review to identify and summarize the currently available evidence-based recommendations.

**Methods:**

We searched MEDLINE, EMBASE, PsycINFO, the Cochrane database of systematic reviews, and websites for clinical guidelines and recommendations. We summarized the type of the publications, developers, approach of health care recommendation development, year and country of publication, and conditions that were addressed. We categorized the direction and strength of each recommendation.

**Results:**

We identified 12 eligible publications. Publication years ranged from 2007 to 2019; four (33.3%) of them were published in 2018. Canada ranked first for the number of publications (*n* = 4, 33.3%). Most (*n* = 11, 92%) of the included recommendations were based on both a systematic review of the best evidence and expert consensus. All the included publications provided a recommendation supporting medical cannabis for CNCP in general and for the specific conditions of neuropathic pain, chronic pain in people living with Human Immunodeficiency Virus (HIV), and chronic abdominal pain, with detailed information sharing and comprehensive consideration of a patient's own values and preferences.

**Conclusion:**

Clinicians can attend to the guidance currently offered, being aware that only weak recommendations are available for medical cannabis in patients with CNCP, as a third- or fourth-line therapy. Detailed discussions with patients regarding the benefits in reducing pain and potential adverse effects are required before its prescription.

## 1. Introduction

There has been a growing interest in the use of cannabinoids in medicine, particularly for the management of chronic pain. It has been shown that delta-9-tetrahydrocannabinol (THC), the principal psychoactive compound of the *Cannabis sativa* plant, may modulate pain perception through its interaction with two cannabinoid receptors: cannabinoid receptor 1 (CB1) and CB2. Exogenous cannabinoids or external cannabinoid receptor agonists proved to be effective in reducing pain, in both animal models and clinical trials [1, 2]. The other major cannabis compound with substantial medical effects is cannabidiol (CBD), which does not influence cognitive function and can actually counteract the psychoactivity of THC. The fact that CBD-rich cannabis is nonpsychoactive or less psychoactive than THC-dominant strains makes it an appealing option for patients looking for symptomatic relief without disconcerting feelings of lethargy or dysphoria. Furthermore, cannabinoids play a positive role in patients' perception and coping of pain by their central mechanism of altering the negative and anxious emotions related to pain [3–5]. Synthetic cannabinoids are full agonists, mostly, at the cannabinoid receptor CB1. They include ajulemic acid, benzopyranoperidine, levonantradol, nabilone, and dronabinol [2].

Chronic noncancer pain (CNCP) is defined as any painful condition that persists for three or more months that is not associated with a diagnosis of cancer. Such conditions include neuropathic pain, low back pain, osteoarthritis, rheumatoid arthritis, fibromyalgia, irritable bowel syndrome, and headache [6]. CNCP interferes with activities of daily living and has a marked negative impact on the quality of life and physical functioning. It is a major cause of morbidity, affecting as much as 30% of the population worldwide [7, 8].

Medical use of cannabis has been the focus of numerous health care recommendations. These statements are intended to optimize patient care and are informed by a systematic review of the evidence providing an assessment of the benefits and harms of alternative care options [9–11]. Recommendations are often developed in the context of clinical practice guidelines and play an important role in facilitating more consistent, effective, and efficient medical practice in order to improve health outcomes [12, 13].

We aimed to search for and summarize all published health care recommendations, including those stated in clinical practice guidelines, to inform clinicians, patients, and policy-makers when considering the use of medical cannabis for CNCP. In this review, we systematically searched and summarized the available recommendations for treating CNCP with medical cannabis.

## 2. Methods

We reviewed and synthesized the evidence following the Cochrane Handbook methodology [14]. We reported our results according to PRISMA (Preferred Reporting Items for Systematic Reviews and Meta-Analyses) recommendations [15].

### 2.1. Eligibility Criteria

We included publications that provided a recommendation on the use of medical cannabis for CNCP, which included clinical practice guidelines, recommendations, consensus statements, position statements, practice statements, and health care standards. The recommendations covered either a single condition or multiple conditions that are considered CNCP. If we identified multiple publications by the same organization, we only included the most recent statement or recommendation.

We excluded publications that were only evidence syntheses, specifically systematic reviews with or without meta-analyses, review articles, and commentaries. Publications in which conditions were not explicitly CNCP (e.g., Crohn's disease not specified as chronic or acute) were excluded.

### 2.2. Data Sources and Search Strategy

We identified relevant publications using a systematic search of MEDLINE, EMBASE, and PsycINFO from the databases' inception to August 28, 2020. Keywords included chronic or intractable or refractory or persistent pain, cannabis, cannabinoids, names and abbreviations of specific cannabis drug names, and recommendation (see the Appendix). We also conducted an open-ended Google search with the keywords: recommendation, consensus, guideline, position statement, practice statement, standard, guide, and “cannab.” We searched the websites of the Cochrane Library (https://www.cochranelibrary.com/), the United States (US) Department of Health and Human Services (http://www.ahrq.gov), the Scottish Intercollegiate Guidelines Network (SIGN) (http://www.sign.ac.uk), the National Institute for Health and Care Excellence (NICE) (http://www.nice.org.uk), the Canadian Medical Association Infobase (http://www.joulecma.ca/cpg), and the Nova Scotia Health authority website list of guidelines (library.nshealth.ca/Cannabis/Clinicians) for relevant clinical practice guidelines.

We checked the references of the included articles for any additional eligible publications.

### 2.3. Publication Selection and Data Abstraction

Two reviewers (YC and MZ) independently examined and selected titles and available abstracts. We retrieved the full text of potentially eligible publications and screened them according to the inclusion and exclusion criteria. We resolved disagreements by discussion.

We created a data abstraction form with Microsoft Excel. One reviewer extracted data; the other double-checked the results. Abstracted data included the organization which made the recommendation, the country, primary condition(s), and/or settings addressed in the recommendation, development approach, funding sources, intended users of the document, and details of the final recommendation.

### 2.4. Data Analysis

We narratively summarized the recommendations on medical cannabis for CNCP. Information on whether patients were involved in the recommendation was based on either one of two factors: (1) if they were included in the panel, regardless of whether or not they voted, or (2) a systematic review exploring patients' values and preferences was conducted [13, 16]. We cited the recommendations from the included publications by a specific condition. We evaluated the strength of recommendations from the included publications by following the GRADE (Grading of Recommendations Assessment, Development, and Evaluation) approach [16, 17]. We classified the strength of each recommendation into one of four categories: (1) strong recommendation for, (2) weak recommendation for, (3) weak recommendation against, or (4) strong recommendation against. Recommendations graded as “strong” should be applied to all or almost all patients, whereas those graded as “weak” should be applied to most patients and require individualization to the patients' values, preferences, and circumstances [13, 17].

We collated the abstracted information and summarized relevant data in tables.

## 3. Results

### 3.1. Literature Search

**Figure 1:**
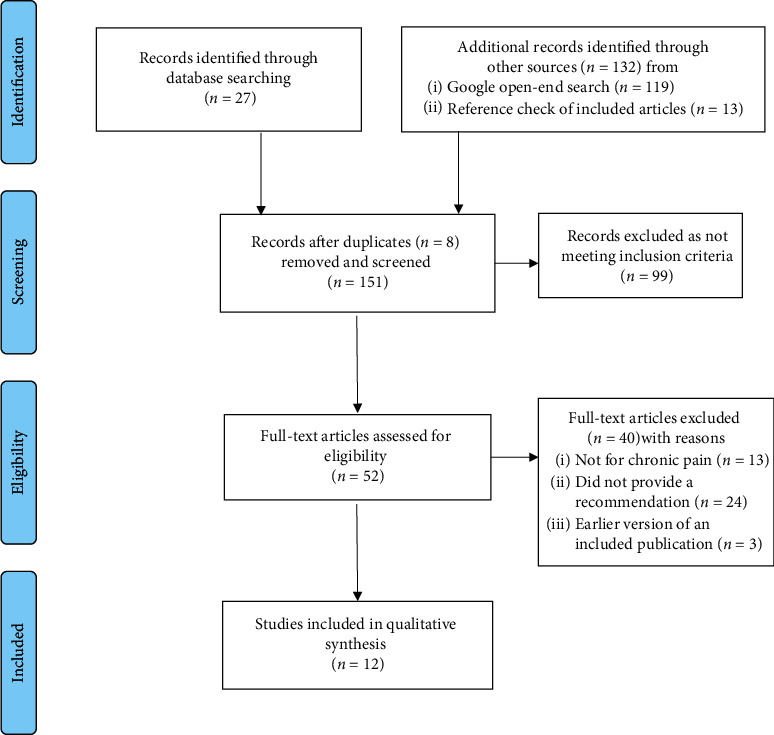
Eligibility assessment PRISMA flow diagram.

We identified 27 references from the electronic database search and 132 references through the other sources. After removing the eight duplicates, we screened 151 references and found 12 to be eligible for our research objective. The PRISMA [15] flowchart of the study selection is presented in Figure 1.

### 3.2. Characteristics of the Included Publications

Of the 12 included publications (Table 1), five were clinical practice guidelines [18, 21, 24–26] and seven were evidence-based recommendations [19, 20, 22] or statements [23, 27–29]. All articles were developed and published by a government entity [20, 21, 25], medical society [19, 23, 24, 26–29], or panel of domain experts [18, 22].

Ten publications were financially supported by a government entity or medical society, of which three reported receiving research grants or honoraria from pharmaceutical companies [18, 19, 23]. One publication was solely financed by an educational grant from a pharmaceutical company [22].

All of the included articles were published between 2007 and 2019. Among these, one-third of them were published in 2018.

**Figure 2:**
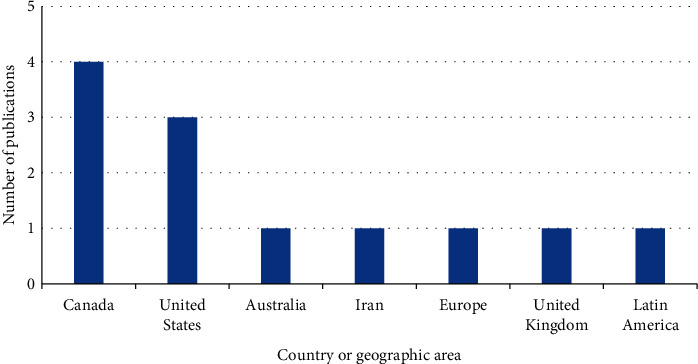
Number of publications by country or geographic area.

Geographically, Canada ranked first in terms of the number of publications that addressed medical cannabis for chronic noncancer pain (*n* = 4 articles by four Canadian societies, 33.3%), followed by the US (*n* = 3, 25.0%). One article was published in each of Australia, Iran, the United Kingdom, and Latin America (involving experts from Chile, Colombia, Ecuador, El Salvador, Honduras, Mexico, Peru, Puerto Rico, and Venezuela) [18]. The European Pain Federation (EFIC) published one article [28] involving experts from Germany, Ireland, the United Kingdom, Finland, Slovenia, Austria, Belgium, France, and Israel (Figure 2). Except for one article, in which the Canadian Rheumatology Association generated the recommendation by consensus from a core group of experts [27], recommendations were developed via a systematic literature review followed by panel discussion and consensus [18–26, 28, 29].

One publication indicated that the intended users of their health care recommendation were both doctors and patients [21], while five others stated that their recommendation was targeted towards health care professionals only [20, 23, 24, 28, 29]. The other six articles did not state the intended users of their recommendation [18, 19, 22, 25–27].

More than half of the included articles provided recommendations on neuropathic pain. Specifically, four articles addressed neuropathic pain associated with multiple sclerosis [18, 19, 22, 25] and three addressed neuropathic pain in general [20, 26, 29]. Two articles were on chronic noncancer pain in general [21, 28]. One article was on chronic pain in patients living with Human Immunodeficiency Virus (HIV) [24]. Lastly, there was one article on rheumatic pain [27] and another on chronic abdominal pain as a gastrointestinal symptom [23].

### 3.3. Direction and Strength of Recommendations

All 12 publications provided weak recommendations supporting medical cannabis for CNCP and were based on current best evidence and expert consensus (Table 1). Based on these recommendations, medical cannabis could be used for treating CNCP in general and for the specific conditions of neuropathic pain, chronic pain in people living with HIV, and chronic abdominal pain.

The justifications for the recommendations included moderate-quality evidence of positive effects with cannabinoids [23, 29], limited benefits and high risk of harms [26], unclear long-term efficacy and safety due to lack of long-term follow-up data [18, 19], and need of further studies to investigate potential drug interactions, as well as efficacy and safety of cannabis for chronic pain [19, 21].

Two publications discussed specific types of cannabis: oral cannabis in Sahraian et al.'s recommendation [22] and cannabis extract, THC, and nabiximols in the American Academy of Neurology (AAN) guideline [25]. The other included articles only addressed medical cannabis in general.

Six publications (50%) explicitly recommended against the use of medical cannabis as a first- or second-line therapy, or as an alternative to standard care [19, 21, 23, 26–29]. Such statements are in accordance with the weak recommendations in favor of cannabis for CNCP established in the majority of the included publications. In other words, consider cannabis as a third-line treatment option if other therapies have failed in pain management. The AAN guideline only recommended medical cannabis for patients with nociceptive (i.e., musculoskeletal) pain from multiple sclerosis, but not for those with neuropathic pain [25]. The NICE guideline stated treating neuropathic pain with medical cannabis only when advised to do so by a pain specialist [20].

We presented the quotes of relevant recommendations from included publications in Table 2.

## 4. Discussion

Recommendations are often seen in guidelines, serve as a summary of the body of evidence on a topic, and are actionable for clinical decision-making [9, 17]. We systematically searched all published recommendations on the use of medical cannabis for treating CNCP. More than half of the identified articles were published in the last three years by authors located in various countries and regions, including Canada, the US, Europe, Australia, Latin America, and Iran. Such global increasing efforts show that the political and cultural backdrop surrounding cannabis has been undergoing a major shift, leading to wider societal acceptance. This also highlights the need for regulation and evidence-based clinical practice guidelines regarding its use.

Currently, the recreational and medical use of cannabis is legal across Canada and in 10 states in the US, with an additional 23 states permitting medical access only [30]. Overall, the greater access to cannabis in North America has led to rapidly increasing interest in the possible benefits and harms surrounding its use. A systematic review and meta-analyses of randomized controlled trials (RCTs) showed that cannabinoids had a statistically significant pain reduction effect compared to placebo among patients with CNCP after a period of treatment of less than 2 weeks (weighted mean difference (WMD) −0.54, 95% confidence interval (CI) −0.76 to −0.31), up to 2 months (WMD −0.68, 95% CI −0.96 to −0.40), and up to 6 months (WMD −0.43, 95% CI −0.75 to −0.10), measured on a 0–10 pain visual analog scale [31]. Furthermore, the movement away from opiates as an analgesic has fuelled an increased interest in applications for cannabinoids in the treatment of chronic noncancer-related pain. It is well documented that cannabis therapy is associated with a significant reduction in opioid requirements [32–34] and, consequently, a reduction in opioid-related adverse effects. Nonsteroidal anti-inflammatory drugs are also commonly used in the treatment of chronic pain [35, 36] but are associated with higher a risk of gastrointestinal events (risk ratio compared to placebo: 1.38, 95% CI 1.21, 1.57) [36, 37] and account for nearly 30% of adverse drug reactions causing hospital admission [38].

Cannabis is associated with central nervous system adverse effects such as psychosis and cognitive impairment and gastrointestinal adverse effects such as dry mouth, nausea, and cannabinoid hyperemesis syndrome, which can impact the quality of life of a patient. Cannabis dependence and addiction are caused by the long duration of cannabis administration and its rewarding effects [23, 28]. The challenges in the use of cannabis include the potential adverse effects of cannabis, the risk of misuse, lacking adequate knowledge and awareness on cannabis, and various limitations on research and formulation [39, 40]. Studies show that there are no deaths or life-threatening side effects associated with the use of cannabis [30, 41]. Clinical practice recommendations cannot account for individual variation among patients (e.g., palliative care versus care of complex regional pain syndrome and spasticity versus abdominal pain).

Current recommendations are consistent as a weak recommendation in favor of medical cannabis as a third- or fourth-line option for CNCP. Based on the GRADE approach, strong recommendations are the practices that can be applied to all or almost all patients in all or almost all circumstances. Weak recommendations are the therapies that require a detailed discussion with patients, often with a shared decision-making process. Patients have to weigh desirable and undesirable effects and comprehensively consider their own values and preferences, as well as cost-effectiveness factors, based on the information and explanation that their physician provides [17].

As per the GRADE framework, recommendations should be made based on the best current evidence. The majority (all except for one) of the included publications developed their recommendation based on a comprehensive systematic review of RCTs. All the included publications involved a multidisciplinary group of individuals with research and clinical expertise relevant to the pathophysiology and management of neuropathic pain. The experts' conceptions and clinical experiences were also fully considered and they reached a consensus.

The GRADE framework also suggests considering two other factors in evaluating the trustworthiness of a recommendation: (1) whether values and preferences associated with the outcomes are appropriately specified and (2) whether the influence of conflicts of interest is minimized [13, 16]. In two of the 12 included publications, the authors reported explicit and transparent consideration or involvement of patients' values and preferences in the recommendation [20, 26]; we were uncertain of this in two of the other publications [23, 24]. The remaining eight publications did not include patients in their panels [18, 19, 21, 22, 25, 27–29]. Of the two recommendations that involved patients [20, 26], both were consistent with the other recommendations (i.e., weak recommendation in favor).

Four of the 12 publications [18, 19, 22, 23] reported joint or independent financial support in the form of honoraria or education grants from pharmaceutical companies. We did not find the recommendations in these publications different from those in the other publications.

The strengths of the present study included explicit eligibility criteria, a comprehensive search for relevant recommendations in all languages, and duplicate assessment of eligibility, data abstraction, and synthesis. A recently published review by the Canadian Agency for Drugs and Technologies in Health (CADTH) summarized findings across relevant systematic reviews and recommendations in guidelines that addressed medical cannabis for the treatment of chronic pain [39]. The authors identified six guidelines, while we conducted a broader search and identified 12 recommendations.

The limitations of our study were primarily related to deficiencies in the reporting of the eligible publications. This is understandable given that medical cannabis has not been legalized in the majority of global regions. Secondly, we did not appraise the quality of the recommendations, due to the lack of a reliable and appropriate assessment tool or criteria to serve such a purpose. The review by CADTH used the AGREE II (Appraisal of Guidelines Research and Evaluation II) tool [42] to address the strengths and limitations of their six included guidelines [39]. Another review of guidelines on neuropathic pain management presented domain scores (the proportions of assessment items fulfilled) for AGREE II [40]. We did not apply AGREE II [42] to our included publications because we not only included guidelines but also included evidence-based recommendations in our systematic review. We discussed the reliability of the recommendations based on the GRADE approach. Finally, while the systematic review of RCTs provides a more measured approach for evaluating the effectiveness of cannabis in the treatment of CNCP, clinical recommendations are mostly general statements and may be in lack of specific guidance on the use of a drug or the supporting evidence.

Several key questions remain unresolved with regard to medical cannabis for the treatment of CNCP. Whether certain types of cannabinoids are superior to others in maintaining benefits with minimum adverse effects, what the optimal doses and routes of administration are, and the impact of accessibility and cost are important factors to consider in the development of recommendations and guidelines. Clearer guidance will require well-designed and large RCTs with reasonable long-term follow-up. The synthesis of currently available clinical practice recommendations suggests that clinicians can attend to the guidance currently offered, being aware that only weak recommendations are available for cannabis in patients with CNCP, as a third- or fourth-line therapy. Detailed discussions with patients regarding benefits in reducing pain and potential adverse effects are required before its prescription.

## Figures and Tables

**Table 1:** Characteristics and summary of recommendations of medical cannabis for chronic pain management.

First author/organization	Publication year	Geographic region	Type of publication	Condition	Direction and strength of recommendation	Additional recommendations
Acevedo [18]	2009	Latin America	Guideline	Neuropathic pain associated with multiple sclerosis	Weak in favor	Not provided
Allan/CFP [26]	2018	Canada	Guideline	Neuropathic pain	Weak in favor	Recommend against as first- or second-line therapy
Australian DoH [21]	2017	Australia	Guideline	Chronic noncancer pain	Weak in favor	Recommend against as first-line therapy
Bruce/HIVMA of IDSA [24]	2017	United States	Guideline	Chronic pain in people living with HIV	Weak in favor	Not provided
Andrews/CAG [23]	2019	Canada	Evidence-based statement	Chronic abdominal pain listed in nonspecified gastrointestinal symptoms	Weak in favor	Recommend against as first-line therapy
CRA [27]	2019	Canada	Evidence-based statement	Rheumatic pain	Weak in favor	Recommend against as an alternative to standard care
Dworkin/IASP [19]	2007	United States	Evidence-based recommendation	Neuropathic pain associated with multiple sclerosis	Weak in favor	Recommend against as first- or second-line therapy
Häuser/EFIC [28]	2018	Europe	Evidence-based statement	Chronic noncancer pain	Weak in favor	Recommend against as first- or second-line therapy
Moulin/CPS [29]	2014	Canada	Evidence-based statement	Neuropathic pain	Weak in favor	Recommend against as first- or second-line therapy
NICE [20]	2013	United Kingdom	Evidence-based recommendation	Neuropathic pain	Weak in favor	Recommend against in nonspecialist settings
Sahraian [22]	2018	Iran	Evidence-based recommendation	Neuropathic pain associated with multiple sclerosis	Weak in favor	Not provided
Yadav/AAN [25]	2014	United States	Guideline	Neuropathic pain associated with multiple sclerosis	Weak in favor	Recommend against for central neuropathic pain

AAN = American Academy of Neurology; Australian DoH = The Australian Government Department of Health; CAG = Canadian Association of Gastroenterology; CFP = Canadian Family Physician; CPS = Canadian Pain Society; CRA = Canadian Rheumatology Association; EFIC = European Pain Federation, formerly the European Federation of IASP Chapters; HIV = Human Immunodeficiency Virus; HIVMA of IDSA = HIV Medicine Association of Infectious Diseases Society of America; IASP = the International Association for the Study of Pain; NICE = the National Institute for Health and Care Excellence.

**Table 2:** Quotes of recommendations from included publications.

First author/organization (year)	Quoted recommendations from publications
Acevedo (2009)	“Cannabinoids can be used for NP (neuropathic pain) associated with multiple sclerosis, but the long-term effects remain unclear”
Allan (2018)	“Neuropathic pain: We recommend against medical cannabinoids as first- or second-line therapy in neuropathic pain owing to limited benefits and high risk of harms (strong recommendation) Clinicians could consider medical cannabinoids for refractory neuropathic pain, with the following considerations (weak recommendation) A discussion has taken place with patients regarding the benefits and risks of medical cannabinoids for pain Patients have had a reasonable therapeutic trial of ≥3 prescribed analgesics and have persistent problematic pain despite optimized analgesic therapy Medical cannabinoids are adjuncts to other prescribed analgesics”
Australian DoH (2017)	“The use of medications, including medicinal cannabis, is not the core component of therapy for CNCP. Cannabinoids should not replace current approved first-line treatments for pain and there is significant potential for drug interactions which needs further study”
Bruce (2017)	“Medical cannabis may be an effective treatment in appropriate patients living with human immunodeficiency virus and chronic pain”
CAG (2018)	“Cannabis and cannabinoids may be helpful for GI symptom control, such as abdominal pain where conventional therapies have failed. […] moderate-quality evidence supported the use of cannabinoids for the treatment of chronic pain […] Medical cannabis use should not replace health Canada approved medical therapy for treatment of any gastroenterologic or hepatologic disease if the approved therapy is available and has not been used”
CRA (2019)	“Medical cannabis is not an alternative to standard care for any rheumatic disease, and rheumatologists should adhere to current treatment standards and guidelines for rheumatic disease management. Common reasons that patients may consider use of medical cannabis are for pain relief, improvement in mood and/or sleep promotion. Current treatment strategies for pain relief and sleep promotion, including non-pharmacologic treatments must be tried before consideration of trial of medical cannabis”
Dworkin (2007)	“Based on the results of a small number of RCTs, the following specific medications should be considered for patients with central NP: […] cannabinoids for NP associated with multiple (was “ple” cut off in the quote?) sclerosis. […] Lack of long-term follow-up data, limited availability, and concerns over precipitating psychosis or schizophrenia, especially in individuals with environmental or genetic risk factors, restrict the use of cannabinoids to second-line therapy for patients with multiple sclerosis NP at present, and additional trials are needed to further establish their efficacy and safety”
Hauser (2018)	“Chronic neuropathic pain: Cannabis‐based medicines can be considered as third‐line therapy. Chronic non-neuropathic non-cancer pain: In exceptional cases, cannabis‐based medicines can be considered as an individual therapeutic trial, if all established treatments have failed and after careful analyses and multidisciplinary assessment”
Moulin (2014)	“One class of medication is recommended for third-line treatment in the management of NeP (neuropathic pain)–cannabinoids”
NICE (2013)	“Do not start the following (including *Cannabis sativa* extract) to treat neuropathic pain in non-specialist settings, unless advised by a specialist to do so”
Sahraian (2018)	“Oral cannabis could be effective for central dysaesthetic pain”
Yadav (2014)	“Clinicians might offer oral cannabis extract for spasticity symptoms and pain (excluding central neuropathic pain) Clinicians might offer tetrahydro-cannabinol (THC) for spasticity symptoms and pain (excluding central neuropathic pain) Clinicians might offer sativex oromucosal cannabinoid spray (nabiximols) for spasticity symptoms, pain, and urinary frequency Data are inadequate to support or refute use of the following in MS: […] smoked cannabis for spasticity, pain, […]”

## Appendix

The MEDLINE title and abstract search strategy:

1. exp Pain/
2. exp Chronic Pain/
3. pain$.mp.
4. (chronic or intractable or refractory or persistent).mp. [mp = title, abstract, original title, name of substance word, subject heading word, floating sub-heading word, keyword heading word, organism supplementary concept word, protocol supplementary concept word, rare disease supplementary concept word, unique identifier, synonyms]
5. 1 or 3
6. 4 and 5
7. 2 or 6
8. fibromyalgia.mp.
9. fibrositis.mp.
10. arthriti$.mp.
11. back pain.mp.
12. neck pain.mp.
13. exp Musculoskeletal Diseases/
14. exp Joint Diseases/
15. exp Back Pain/
16. exp Multiple Sclerosis/
17. multiple sclerosis.mp.
18. allodynia.mp.
19. sciatic$.mp.
20. neuralgia.mp.
21. neuropath$.mp.
22. 7 or 8 or 9 or 10 or 11 or 12 or 13 or 14 or 15 or 16 or 17 or 18 or 19 or 20 or 21
23. cannabis.mp.
24. exp Cannabis/
25. cannabinoid$.mp.
26. exp Cannabinoids/
27. tetrahydrocannabinol.mp.
28. exp Dronabinol/
29. cannabidiol.mp.
30. exp Cannabidiol/
31. sativex.mp.
32. medical marijuana.mp. or Medical Marijuana/
33. nabiximols.mp.
34. thc.mp.
35. nabilone.mp.
36. cesamet.mp.
37. marinol.mp.
38. marihuana.mp.
39. marijuana.mp.
40. hashish.mp.
41. 23 or 24 or 25 or 26 or 27 or 28 or 29 or 30 or 31 or 32 or 33 or 34 or 35 or 36 or 37 or 38 or 39 or 40
42. 22 and 41
43. guideline.m_titl
44. recommendation.m_titl
45. practice statement.m_titl
46. 43 or 44 or 45
47. 42 and 46.
